# Viral gut metagenomics of sympatric wild and domestic canids, and monitoring of viruses: Insights from an endangered wolf population

**DOI:** 10.1002/ece3.2991

**Published:** 2017-04-27

**Authors:** Nádia Conceição‐Neto, Raquel Godinho, Francisco Álvares, Claude K. Yinda, Ward Deboutte, Mark Zeller, Lies Laenen, Elisabeth Heylen, Sara Roque, Francisco Petrucci‐Fonseca, Nuno Santos, Marc Van Ranst, João R. Mesquita, Jelle Matthijnssens

**Affiliations:** ^1^ Department of Microbiology and ImmunologyLaboratory of Viral MetagenomicsRega Institute for Medical ResearchKU Leuven – University of LeuvenLeuvenBelgium; ^2^ Department of Microbiology and ImmunologyLaboratory of Clinical VirologyRega Institute for Medical ResearchKU Leuven – University of LeuvenLeuvenBelgium; ^3^ CIBIO/InBIOCentro de Investigação em Biodiversidade e Recursos GenéticosUniversidade do PortoVairãoPortugal; ^4^ Departamento de BiologiaFaculdade de CiênciasUniversidade do PortoPortoPortugal; ^5^ cE3c, Centre for Ecology, Evolution and Environmental ChangesFaculdade de Ciências da Universidade de LisboaLisbonPortugal; ^6^ Departamento de Biologia AnimalGrupo LoboFaculdade de Ciências da Universidade de LisboaLisbonPortugal; ^7^ Department of Zootechnics, Rural Engineering and VeterinaryAgrarian Superior School of ViseuViseuPortugal

**Keywords:** bocavirus, canine distemper virus, *Canis lupus signatus*, conservation, noninvasive sampling, viral metagenomics

## Abstract

Animal host–microbe interactions are a relevant concern for wildlife conservation, particularly regarding generalist pathogens, where domestic host species can play a role in the transmission of infectious agents, such as viruses, to wild animals. Knowledge on viral circulation in wild host species is still scarce and can be improved by the recent advent of modern molecular approaches. We aimed to characterize the fecal virome and identify viruses of potential conservation relevance of diarrheic free‐ranging wolves and sympatric domestic dogs from Central Portugal, where a small and threatened wolf population persists in a highly anthropogenically modified landscape. Using viral metagenomics, we screened diarrheic stools collected from wolves (*n* = 8), feral dogs (*n* = 4), and pet dogs (*n* = 6), all collected within wolf range. We detected novel highly divergent viruses as well as known viral pathogens with established effects on population dynamics, including canine distemper virus, a novel bocavirus, and canine minute virus. Furthermore, we performed a 4‐year survey for the six wolf packs comprising this endangered wolf population, screening 93 fecal samples from 36 genetically identified wolves for canine distemper virus and the novel bocavirus, previously identified using our metagenomics approach. Our novel approach using metagenomics for viral screening in noninvasive samples of wolves and dogs has profound implications on the knowledge of both virology and wildlife diseases, establishing a complementary tool to traditional screening methods for the conservation of threatened species.

## 1 Introduction

Infectious diseases may have direct effects on demographic patterns of wildlife populations and are seen as a substantial threat to the conservation of global biodiversity (Daszak, Cunningham, & Hyatt, 2000; Gordon et al., 2015). Viruses remain a major cause of disease outbreaks in wildlife, partially due to anthropogenic effects. Disease surveillance is of particular importance for wild canids, as their evolutionary proximity to domestic dogs increases the risk of disease spill over (Bryan et al., 2011; Pedersen, Jones, Nunn, & Altizer, 2007). In fact, domestic dogs are recognized as an important risk factor for disease in sympatric wild canids, including wolves (*Canis lupus*), as dogs usually attain much higher population size compared to wild canids (Bryan et al., 2011; Cleaveland, Kaare, Knobel, & Laurenson, 2006; Lescureux & Linnell, 2014). Epidemics of diseases, such as canine distemper and canine parvoviruses, can severely affect wolf population dynamics. Canine distemper was suggested to cause high pup mortality (60%–90%) and periodic wolf population declines in the Greater Yellowstone area (Almberg, Cross, Dobson, Smith, & Hudson, 2012; Almberg, Cross, & Smith, 2010; Almberg, Mech, Smith, Sheldon, & Crabtree, 2009). Canine parvovirus caused a catastrophic decline in the wolf population in Isle Royale (Wilmers, Post, Peterson, & Vucetich, 2006) and was shown to reduce wolf recruitment by 40%–60% in Minnesota (Mech, Goyal, Paul, & Newton, 2008). The transmission of infectious diseases between domestic dogs and free‐ranging wolves has also been documented in human‐dominated landscapes in Portugal (Müller et al., 2011). However, due to lack of appropriate studies, there is only very limited data available on these and other viral pathogens that might be circulating in wild canid populations.

Next generation sequencing (NGS) revolutionized the way we study ecosystems, as it offers a broad, noninvasive tool to detect nucleic acids from different types of pathogens, depending on the sample type and preparation method used. In the virology field, viral detection is traditionally performed using classical methods such as antigen‐, antibody‐, or nucleic acids‐detection assays targeting a specific agent. However, the recent democratization of NGS technologies allows researchers to perform diagnostics without targeting, a priori, specific infectious agents. Its high sensitivity allows the detection of pathogens present at very low abundance in biological samples. This approach is particularly suited to study the occurrence of known and unknown viruses in wildlife populations, coupled with noninvasive sampling methods. Furthermore, this is particularly relevant for free‐ranging wolves from which is exceedingly difficult to obtain fresh biological samples, due to their elusive nature.

Wolves in the Iberian Peninsula (*Canis lupus signatus*) occur in a heterogeneous and anthropogenically modified landscape, coping with intense human‐related factors of disturbance, such as settlements, infrastructures, or domestic dogs, but also profiting from food sources such as livestock (Eggermann, da Costa, Guerra, Kirchner, & Petrucci‐Fonseca, 2011; Llaneza, López‐Bao, & Sazatornil, 2012). In Portugal, wolf populations suffered a drastic decline in range and numbers during the 20th century, with the most critical situation being observed in the region located south of the Douro river. The South Douro wolf population suffered a drastic decline on range and numbers in the last few decades persisting in a highly anthropogenically modified landscape at the very edge of the species distribution (Pimenta et al., 2005). Currently, this population faces several threats such as depleted genetic diversity, low prey availability, habitat fragmentation, and geographic isolation from the main Iberian wolf range (Grilo, Moço, Cândido, Alexandre, & Petrucci‐Fonseca, 2002; Hindrikson et al., 2016; Pimenta et al., 2005). Moreover, the few packs comprising this population show low reproduction rates, possibly due to human disturbance in breeding sites, intensive human persecution, and other factors, such as food scarcity and diseases prone to induce pup mortality (Hindrikson et al., 2016; Pimenta et al., 2005; Roque, Bernardo, Godinho, Petrucci‐Fonseca, & Álvares, 2013). As a result, this small wolf population is one of the few in Europe considered to be on the verge of extinction (Boitani & Ciucci, 2009). Furthermore, wolves from this region are highly dependent on human‐related food resources, with livestock comprising over 90% of their diet (Torres & Fonseca, 2016; Vos, 2000). Anthropogenic food sources including extensively bred livestock (cattle, sheep, and goat) or scavenging on carcasses of intensively farmed animals (horses, pigs, rabbits, and poultry; Roque, Palmegiani, Petrucci‐Fonseca, & Álvares, 2012) might increase contacts with domestic dogs and potential for disease spill‐over. This human‐dominated environment, together with demographic isolation and limited genetic diversity, make the occurrence of disease a particularly important conservation issue in this wolf population (Wilmers et al., 2006).

In this study, we aimed to get insights on the occurrence, sharing, and evolutionary relationship of enteric viruses in sympatric wolves and dogs, by characterizing the fecal virome of diarrheic dogs and wolves from the south of Douro region using viral metagenomics. Furthermore, we carried out a PCR screening for bocavirus and canine distemper virus (CDV) on 93 fecal samples covering all six packs occurring in the region. Samples were collected from 2009 to 2012, corresponding to 36 genetically identified individual wolves (Roque et al., 2013).

## 2 Materials and Methods

### 2.1 Sample collection and preparation for viral metagenomics

**Figure 1:**
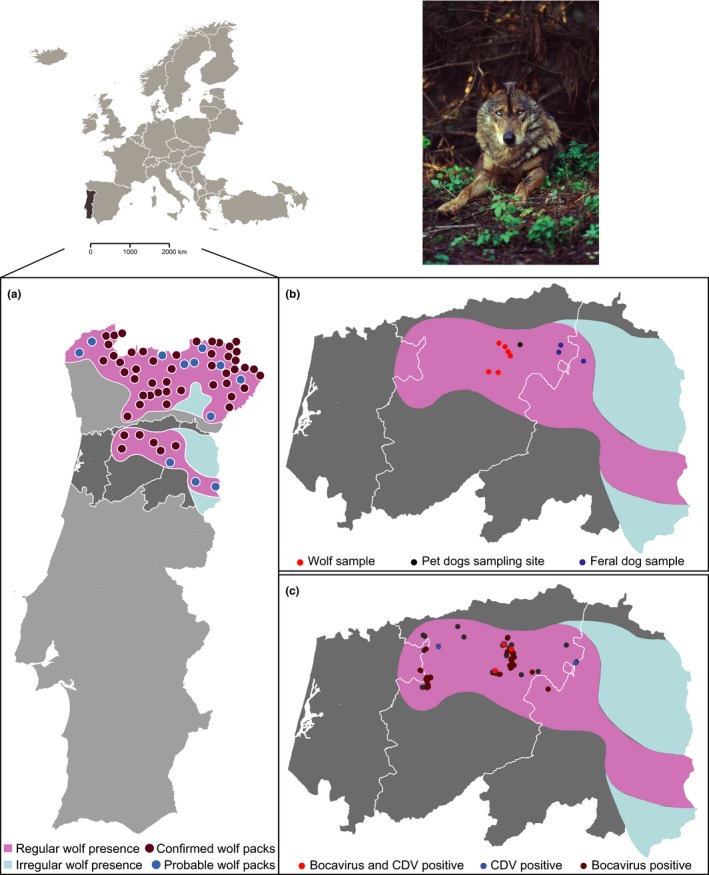
Model organism from the South Douro population and location of sample collection of the diarrheic feces of sympatric wild and domestic canids analyzed in this study. (a) Wolf distribution and known packs in Portugal according to 2002/2003 National Census (Pimenta et al., 2005): regular presence (pink area) and irregular presence (blue area). The northern and central regions of wolf presence are separated by the Douro river, which acts as an isolating barrier. (b) Fecal samples analyzed in the metagenomics study of Iberian wolves (red dots), feral dogs (blue dots), and pet dogs sampled in a veterinarian clinic (black dot) in Central Portugal. (c) Fecal samples analyzed by PCR screening from six packs comprising the endangered wolf population located at the South of Douro region in Central Portugal and collected from 2009 to 2012 (Fig. S1. Wolf photo credits to Artur Oliveira (Centro de Recuperação do Lobo Ibérico)

To investigate putative disease causing agents, diarrheic stools attributed to free‐ranging wolves (*n* = 8), feral dogs (*n* = 4), and pet dogs (*n* = 6) were collected from the Portuguese South of Douro wolf population in the districts of Viseu and Guarda, Portugal in 2011 (Figures 1 and S1). Fecal wolf‐like samples were collected along transects in areas of known occurrence of wolf packs and analyzed for species identification by amplification of a mitochondrial DNA fragment (mtDNA) as previously described (Godinho et al., 2015). Samples of pet dogs were collected at nearby veterinary clinics from owners of urban dogs that reported to occasionally take their animals to areas within wolf range. We use the term “feral” for dog samples collected on the field within wolf range, while “pet” refers to samples of urban dogs collected in veterinary clinics. Diarrheic stools were defined as nonformed, watery feces, after evaluation by a veterinarian. All fecal samples used for viral metagenomics were stored at −20°C before shipping to the laboratory of viral metagenomics (KU Leuven) where they were kept at −80°C until further processing.

Fecal samples were pooled per group: two wolf pools of four samples each, one feral dog pool of four samples, and two pet dog pools of three samples each. Pools were prepared using the NetoVIR protocol (Conceição‐Neto, Zeller, Lefrère, et al., 2015). Fecal material was weighed (≈100 mg) and diluted with PBS to attain 10% (m/v) suspensions. Briefly, ten percent fecal suspensions were homogenized for 1 min at 3000 rpm with a MINILYS homogenizer (Bertin Technologies), briefly centrifuged at 17000 *g* per 3 min and filtered through 0.8 μm filter (Sartorious). The filtrate was then treated with a cocktail of Benzonase (Novagen) and Micrococcal Nuclease (New England Biolabs) at 37°C for 2 hr to digest free‐floating nucleic acids, in a homemade buffer (1 M Tris, 100 mM CaCl_2_, and 30 mM MgCl_2_). RNA and DNA were extracted using the QIAamp Viral RNA Mini Kit (Qiagen) according to the manufacturer's instructions but without addition of carrier RNA to the lysis buffer. First and second strand synthesis and random PCR amplification for 17 cycles were performed using a slightly modified Whole Transcriptome Amplification 2 (WTA2) Kit procedure (Sigma‐Aldrich), with a denaturation temperature of 95°C instead of 72°C to allow for the denaturation of dsDNA and dsRNA. This modification led to the amplification of both RNA and DNA. WTA2 products were purified with MSB Spin PCRapace spin columns (Stratec) and were prepared for Illumina sequencing using the Nextera XT DNA library preparation kit (Illumina), with 4‐min tagmentation time and 45‐s PCR extension. Libraries were quantified with the KAPA Library Quantification kit (Kapa Biosystems), and sequencing of the samples was performed on a HiSeq 2500 platform (Illumina) for 300 cycles (150‐bp paired ends).

### 2.2 Genomic and phylogenetic analysis

Raw Illumina reads were trimmed for quality and adapters using Trimmomatic (Bolger, Lohse, & Usadel, 2014), bacterial reads were removed using Kraken (Wood & Salzberg, 2014), and the remaining reads were de novo assembled into contigs using SPAdes assembler (Bankevich et al., 2012). Contigs were classified using DIAMOND in sensitive mode (Buchfink, Xie, & Huson, 2015). Open reading frames (ORF) were identified with ORF Finder analysis. Amino acid alignments of the viral sequences were performed with MUSCLE (Edgar, 2004). Maximum likelihood phylogenetic trees were constructed in MEGA6.0 (Tamura, Stecher, Peterson, Filipski, & Kumar, 2013), where the best model of substitution was calculated, with 500 bootstrap replicates.

### 2.3 Screening of lupine bocavirus and canine distemper virus in wolf pack feces

We screened 93 fecal samples collected between February 2009 and July 2012, corresponding to 36 individually identified wolves belonging to six packs in South of Douro (Roque et al., 2013), for lupine bocavirus and canine distemper virus. For virus detection, feces were subjected to a DNA extraction following Frantz and colleagues (Frantz et al., 2003). Potential PCR inhibitors were removed after DNA extraction using prerinsed Microcon® YM‐30 Filter Units (Millipore). Negative controls were included throughout the process. Primers were designed for the identified strains, and RT‐PCR was carried out for CDV and PCR for bocavirus. Primer sequences for CDV forward is 5′ – ACT TCC GCG ATC TCC ACT GG – 3′ and reverse is 5′ – GCT CCA CTG CAT CTG TAT GG – 3′. The bocavirus forward primer is 5′ – AGA CCA GAT GCT CCA CAT GG – 3′ and reverse 5′ – TGC CTG CCA CGG ATT GTA CC – 3′. For CDV amplification, an initial reverse transcription step at 50°C for 30 min was followed by a PCR activation step at 95°C for 15 min, 35 cycles of amplification (30 s at 94°C, 30 s at 57°C, and 1 min at 72°C), and a final extension step for 10 min at 72°C in a Biometra T3000 thermocycler (Biometra). For bocavirus amplification, the same PCR settings were used except the initial reverse transcription was omitted. Three PCR replicates were performed to decrease the number of negative results due to the low quantity DNA and RNA in samples. All (RT‐)PCR reactions were performed using the OneStep RT‐PCR kit (Qiagen) in a Biometra T3000 thermocycler (Biometra). The positive samples were purified with ExoSAP‐IT (Affymetrix), and Sanger sequenced with the ABI Prism 3130xl Genetic Analyser (Applied Biosystems). The chromatogram sequencing files were inspected with Chromas 2.3.

## 3 Results

### 3.1 Fecal virome of sympatric wolves and domestic dogs using NGS

After quality trimming and removal of bacterial reads, a total of 18,767,912 reads from the five pools were assembled into contigs and classified using DIAMOND (Buchfink et al., 2015). In total, 2,868,974 sequences (15%) were classified as viral, of which the majority could be attributed to bacteriophage sequences (54%). In fact, members of the order *Caudovirales* and *Microviridae* family were detected in all samples (Table 1). Furthermore, sequences from the following families of viruses that infect eukaryotes were detected in the wolf pools: *Parvoviridae*,* Picornaviridae*,* Circoviridae*,* Mycodnaviridae*,* Picobirnaviridae,* and *Paramyxoviridae* (Table 1). In the feral dog pool, sequences from the *Nodaviridae, Mimiviridae, Totiviridae,* and *Parvoviridae* were detected. In the pet dog pools, *Parvoviridae*,* Mycodnaviridae,* and *Virgaviridae* sequences were detected (Table 1).

**Table 1:** Viruses detected in this study by metagenomics analysis (NGS) of fecal samples from Iberian wolves and domestic dogs in South of Douro region

	Viral family	Feral dog pool	Wolf pool 1	Wolf pool 2	Pet dog pool 1	Pet dog pool 2
Number of reads blasted		4,003,381	3,272,882	2,874,956	4,501,170	4,115,523
Number of viral reads		939,118	911,359	417,65	350,114	250,733
Bacteriophage reads	*Caudovirales* and *Microviridae*	580,772	606,791	136,777	5464	208,79
Densovirus^a^	*Parvoviridae*		6876	206,174		
Parvovirus^a^	*Parvoviridae*		7279	28		
Dependoparvovirus	*Parvoviridae*	3990				
Fesa‐like virus	Unassigned		265	81		1573
Kobuvirus	*Picornaviridae*		189			
Nodavirus^a^	*Nodaviridae*	293				
Mimivirus	*Mimiviridae*	742				
Totivirus	*Totiviridae*	1051	431			
Circularviruses	*Circoviridae*		1,013,048	28757		
Bocavirus^a^	*Parvoviridae*		25,418		342,576	
Canine distemper virus^a^	*Paramyxoviridae*		1130			
Gemycircularvirus^a^	*Mycodnaviridae*		103		494	
Picobirnavirus^b^	*Picobirnaviridae*		67			
Negevirus	Unassigned			591		
Tobamovirus	*Virgaviridae*					23,956

a Virus characterized in further detail in this manuscript.

b Reported elsewhere (Conceição‐Neto, Mesquita, et al., 2016).

### 3.2 Identification of canine distemper virus in wolf samples using NGS

The genus *Morbillivirus,* belonging to the family *Paramyxoviridae*, includes several known pathogens such as measles and CDV. Viruses belonging to the family *Paramyxoviridae* have negative strand single‐stranded RNA genomes of 15 kb and encode six proteins. The hemagglutinin (H) protein constitutes the envelope glycoprotein spikes on the virion and allows for host cell entry by attachment to the signaling lymphocytic activation molecule of the host (Ke et al., 2015). In addition, amino acid substitutions in the H gene (positions 530 and 549) likely play a role in host switches (Sekulin et al., 2011). We identified CDV in one of the wolf pools and phylogenetic analysis of the complete H protein gene sequence showed its highest nucleotide similarity (98.0%) with a strain isolated from dogs that died in a 1991 epidemic in Copenhagen and adapted to Vero cells (Nielsen, Andersen, Jensen, Blixenkrone‐Møller, & Bolt, 2003). Positions 530 and 549 of the H protein of our strain revealed the presence of a glycine (G) and a tyrosine (Y), respectively, which is typically found in viral strains identified in dogs (Müller et al., 2011).

### 3.3 Identification of two bocaviruses in wolf and dog using NGS

The *Parvoviridae* is a ssDNA viral family that can be divided into two subfamilies: the *Parvovirinae* which infect vertebrates and the *Densovirinae* infecting arthropods. The *Parvovirinae* subfamily encloses the genus *Bocaparvovirus* (Cotmore et al., 2014). Bocaviruses have been identified in a wide range of hosts so far, such as humans, dogs, gorillas, cats, sea lions, and pigs (Zhou, Sun, & Wang, 2014).

**Figure 2:**
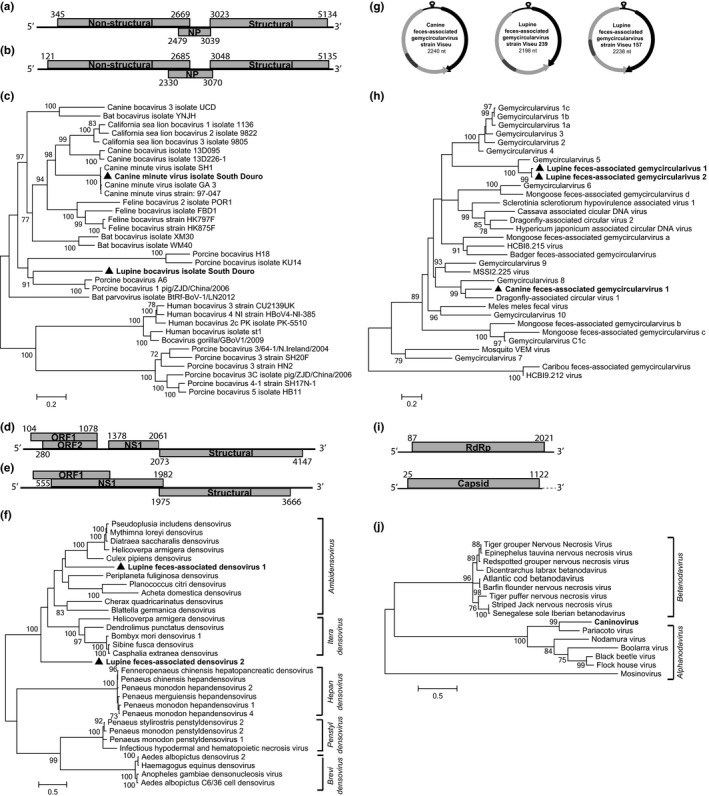
(a) Genome organization of the canine minute virus. (b) Genome organization of the lupine bocavirus. (c) Maximum‐likelihood phylogenetic tree of the aa VP1 sequences of bocaviruses. (d) Genome organization of the lupine feces‐associated densovirus 1. (e) Genome organization of the Lupine feces‐associated densovirus 2. (f) Maximum‐likelihood phylogenetic tree of the aa NS1 sequences of densoviruses. (g) Genome organization of undescribed gemycircularviruses. Depicted in gray is the Rep with an intron and depicted in black is the capsid protein. (h) Maximum‐likelihood phylogenetic tree of the aa replicase sequences of gemycircularviruses. (i) Genome organization of the caninovirus showing a complete RNA1 segment and a partial RNA2. (j) Maximum‐likelihood phylogenetic tree of the aa RNA2 (Capsid) gene sequences of nodaviruses. All trees were built in MEGA6 using 500 bootstraps, and only bootstrap values even or >70 are shown. Strain identified in this study is shown in bold with a triangle

We identified a near complete canine minute virus (CnMV) genome in one pool of diarrheic pet dogs (Figure 2a). Formerly known as canine parvovirus type 1, this virus was renamed and assigned to the *Bocaparvovirus* genus. The complete coding region of a undescribed CnMV isolate South of Douro (5,196 nt), shared 98.9% nucleotide identity with isolate GA 3 isolated from dogs in the USA in 1991 (Sun et al., 2009). Analysis of the VP1 protein shows high conservation and 100% amino acid similarity with isolate SH1 detected in dogs from China (Figure 2c).

We could retrieve a second complete bocavirus coding sequence from a fecal wolf pool (Figure 2b), representing the first bocavirus isolated from a wolf. We named it Lupine bocavirus isolate South of Douro (5,228 nt). Analysis of the structural coat protein of this lupine bocavirus showed low similarity with known bocaviruses and was most similar (62.5% amino acid similarity) to a porcine bocavirus isolated in China (Cheng et al., 2010), forming a distinct and novel cluster among bocaviruses (Figure 2c).

### 3.4 Identification of densoviruses in wolf using NGS

We could detect another two viruses from the *Parvoviridae* family in wolves. This family can be subdivided into two subfamilies: *Parvovirinae* and *Densovirinae*. Members of the first subfamily infect vertebrates, whereas from the latter one infect arthropods, and these ssDNA viruses encode for nonstructural and structural genes in a mono‐ or ambisense genome (Cotmore et al., 2014). Interestingly, 206,174 reads in a wolf were attributed to a densovirus: Lupine feces‐associated densovirus strain Viseu 1 (2D). In addition, in a different wolf sample, another densovirus could be identified. Phylogenetic analysis of the nonstructural gene (NS1) revealed that the Lupine strain 1 clustered among the *Ambidensovirus* genus (Figure 2f), sharing its highest amino acid similarity of 52.1% with the *Helicoverpa armigera* densovirus, isolated from a moth. The latter virus isolated from the other wolf sample formed a separate highly distinct clade and did not cluster among other densoviruses, sharing only 41.7% similarity with *Bombyx mori* densovirus 1, which belongs to the genus *Iteradensovirus* (Figure 2d,f).

### 3.5 Identification of undescribed divergent gemycircularviruses in dog and wolf samples using NGS

Recently a novel family *Mycodnaviridae* and genus *Gemycircularvirus* were proposed for a group of ssDNA circular viruses, of which the first was originally isolated from fungi (Yu et al., 2010). Followed by this discovery, gemycircularviruses have been isolated in a diverse range of hosts, such as cassava plant (Dayaram et al., 2012), dragonflies (Rosario et al., 2012), New Zealand mammals and birds (Sikorski, Dayaram, & Varsani, 2013), feces from mammalian carnivores, namely mongoose (Conceição‐Neto, Zeller, Heylen, et al., 2015) and badger (van den Brand et al., 2012; Conceição‐Neto, Zeller, Heylen, et al., 2015), human feces and sewage (Phan et al., 2015), and human blood samples (Uch et al., 2015; Zhang et al., 2016). We identified three complete gemycircularviruses (Figure 2g), one from a domestic dog (Canine feces‐associated gemycircularvirus 1 strain South of Douro), and two other from wolf (Lupine feces‐associated gemycircularvirus 1 and 2 strain South of Douro). The lupine strain 1 replicase sequence shares 99.7% nucleotide identity with the lupine strain 2. The canine feces‐associated gemycircularvirus was most similar (66.7% of amino acid) to dragonfly‐associated circular virus 1 (Figure 2h).

### 3.6 Identification of caninovirus, an undescribed nodavirus in a feral dog using NGS

The *Nodaviridae* family is divided into two distinct genera: the *Alphanodavirus* and *Betanodavirus*. These viruses are of segmented single‐stranded RNA and the two segments encode for an RdRp (RNA1) of 3.2 kb and a capsid segment of 1.2 kb (RNA2; Ball, Wohlrab, & Li, 1994). Alphanodaviruses have been mostly isolated from insects, opposing to betanodaviruses which infect fish and are responsible for viral nervous necrosis in several species (Bailey & Scott, 1973; Shetty, Maiti, Shivakumar Santhosh, Venugopal, & Karunasagar, 2012). We tentatively named the virus caninovirus (Canine nodavirus), as previously done for Mosinovirus virus (mosquito nodavirus; Schuster et al., 2014; 2I). The capsid of caninovirus shares its highest similarity with the Pariacoto virus (63.3% aa), which was isolated from a southern armyworm in Peru (Figure 2j). Interestingly, the RNA1 of caninovirus shares its highest similarity with the recently identified Lunovirus in a Portuguese otter (60.7% aa; Conceição‐Neto et al. 2015).

### 3.7 PCR screening for canine distemper virus and lupine bocavirus in wolf pack feces

Based on our findings using NGS, a further assessment on the prevalence of canine distemper virus (CDV) and lupine bocavirus in fecal samples from the wolf population was carried out (Figure 1b, Table S1). We selected these two viruses for screening in the endangered South of Douro wolf population as CDV is an established viral pathogen in wolves, and more information of the novel lupine bocavirus is needed to unravel its epidemiology. From the 93 fecal samples corresponding to 36 individual wolves from six packs, nine were PCR positive for CDV and 34 positive for lupine bocavirus, which we further confirmed with Sanger sequencing (Table 2).

**Table 2:** Screening of CDV and lupine bocavirus in wolf fecal samples covering all packs comprising the endangered South of Douro population

	Samples collected	CDV positives	% CDV positives	Bocavirus positives	% Bocavirus positives	Number of wolves positive for CDV	Number of wolves positive for bocavirus
2009	21	0	0.00	6	28.57	0/10	4/10
2010	22	2	9.09	9	40.91	2/10	5/10
2011	35	6	17.14	13	37.14	5/21	7/21
2012	15	1	6.67	6	40.00	1/9	4/9
Total	93	9	9.68	34	36.56	8/36	13/36

**Figure 3:**
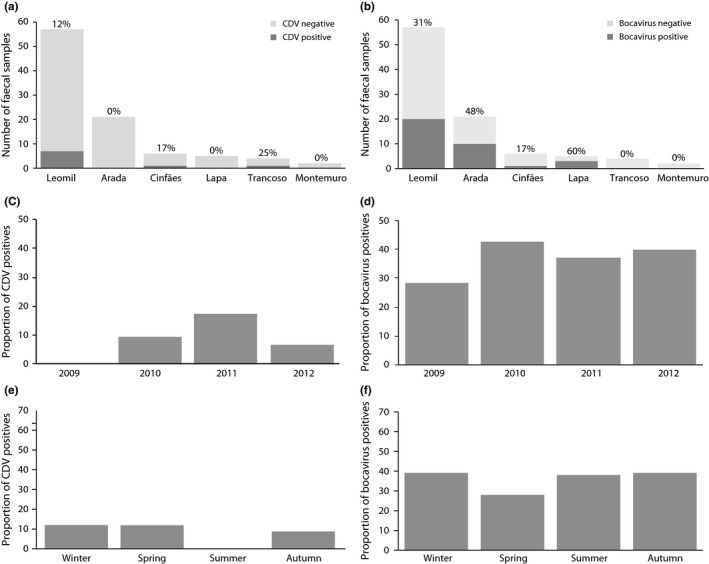
Results of the molecular survey for canine distemper virus (CDV) and bocavirus in fecal samples. Number of positive and negative fecal samples by wolf pack for CDV (a) and bocavirus (b). Proportion of positives by year with over 15 samples analyzed for CDV (c) and bocavirus (d). Proportion of positives by season for CDV (e) and bocavirus (f)

For CDV, the nine positive samples belong to eight different wolves genetically identified, as the same individual was positive twice for the virus in October and December 2011 (Table S1). Two of the positive samples were from 2010, six from 2011, and one from 2012 (Table 2 and Figure 3c). Also, seven of the positive samples were from individuals belonging to the Leomil pack, one from Cinfães pack, and one from Trancoso pack (Table S1 and Figure 3a).

Concerning the lupine bocavirus, the screening of these 93 samples revealed that 34 (36.56%) were PCR positive for the undescribed lupine bocavirus (Table 2). These 34 positive samples correspond to 13 different wolves, and since the same animals were sampled along 4 years (2009–2012), the same single wolf harbored the virus in different occasions (Table 2 and S1). Of note, one of the wolves from which nine samples were available, exhibited seven positive samples for bocavirus over a period of 3 years (2009–2012; Table S1).

Figure 3 enlightens the number of positives per wolf pack for bocavirus and CDV (Panel a and b). Trancoso pack showed the highest CDV positive percentage, but only four samples were collected. An overall overview of the bocavirus and CDV positives per year is represented in Figure 3c,d. Finally, a seasonal outline is also shown (Figure 3e,f). There is a tendency for bocavirus to be detected at similar proportions every year and across seasons, while CDV detection peaks in 2011 and was not detected in 2009, neither during summer.

## 4 Discussion

We applied next‐generation sequencing (NGS) technologies to fecal samples of sympatric endangered wolves, feral dogs, and pet dogs with diarrhea, aiming to study their gut virome, and the potential spread of dog viruses to the endangered South of Douro wolf population. Furthermore, we identified in wolf samples using NGS, canine distemper virus (CDV), a well‐established wolf pathogen and a novel lupine bocavirus, from the *Parvoviridae* family that harbors canine pathogens. Therefore, we performed a wider PCR screening for these two viruses on fecal samples from genetically identified wolves, belonging to the six packs comprising this endangered wolf population in Portugal.

Using NGS, we were able to detect both undescribed and known viruses in wolves and dogs. CDV was identified in feces of wolf and is known to induce high mortality in wild and domestic carnivores (Almberg et al., 2010; Beineke, Baumgärtner, & Wohlsein, 2015). On the Iberian Peninsula, few studies have investigated exposure of free‐ranging wolves to CDV by serological methods, reporting from 0 up to 18.7% seroprevalence (Millán et al., 2016; Santos, Almendra, & Tavares, 2009; Sobrino, Arnal, Luco, & Gortázar, 2008). Using molecular methods, Millán et al. (2016) did not detect CDV in feces and tissue samples from 54 free‐ranging wolves. Müller and colleagues were able to retrieve a complete H protein gene (encoding the viral hemagglutinin, which is responsible for host recognition) from two wolves whose death was attributed to canine distemper, one of which originated in 2008 in the same South of Douro wolf population sampled in our study. Their H protein analyses revealed the presence of a glycine (G) and a tyrosine (Y) at positions 530 and 549, respectively (Müller et al., 2011). These substitutions were also present in the strain from this study and are typically found in CDV strains identified in dogs, suggesting the occurrence of cross‐species transmission events. In other wildlife species, different amino acids have been found at these sites, suggesting a functional role of residue 549 in host switches (McCarthy, Shaw, & Goodman, 2007; von Messling et al., 2005; Sekulin et al., 2011). However, it is possible that mutations at these sites are not required due to the genetic proximity between dogs and wolves (Müller et al., 2011). Our results further support that the CDV strains infecting wolves in Portugal are shared with dogs and may be able to circulate in the wolf population for a prolonged time, confirming that dogs can act as reservoirs of pathogens for wolves, as already suggested previously (Bryan et al., 2011; Müller et al., 2011).

To our knowledge, this is the first survey for CDV in Portuguese wolves using noninvasive fecal sampling, further establishing a reliable new tool for CDV surveillance in this species, as previously carried out in Spain (Millán et al., 2016) or for parvoviruses in Yellowstone National park (Mech, Almberg, Smith, Goyal, & Singer, 2012). Although shedding of CDV is thought to occur mainly in oronasal exudates, the virus replicates widely in lymphoid and epithelial tissues, including the gastrointestinal system (Deem, Ph, Dipl, Spelman, & Dipl, 2000; Williams & Barker, 2008), so fecal shedding is not unexpected. However, it cannot be excluded that some wolves harbored the virus but did not shed it in their feces. As viral shedding is limited to up to 90 days postinfection (Deem et al., 2000), the detection of CDV RNA in 9.8% of the fecal samples, corresponding to 21.6% of the sampled individual wolves from 2009 to 2012, suggests a high exposure to the virus in South Douro wolf population. Nevertheless, the small population size estimated in 18–50 individuals; (Pimenta et al., 2005) is probably not capable of maintaining CDV, requiring spill over from sympatric host species such as dogs or other carnivores, as previously reported to occur (Müller et al., 2011). To further support this fact, the wolves sampled for this study show very reduced movements between packs, being the Arada pack the one reported to be the most isolated (Roque et al., 2013). Moreover, our data show a peak in CDV detection in fecal samples collected in 2011, while in 2009, it was not detected, suggesting an epidemic pattern of infection. This same epidemiological scenario was suggested to occur in the Yellowstone ecosystem (Almberg et al., 2010). Interestingly, in 2011, none of the wolf packs in this population had evidences of breeding occurrence, in contrast with the previous years (2007–2009; Roque et al., 2013), which may suggest an influence of CDV episodes with breeding, by leading to high pup mortality and contributing to the low reproduction rates in this wolf population.

Interestingly, in the same wolf fecal pool where CDV was identified, a complete undescribed lupine bocavirus could be retrieved. This is the first bocavirus isolated from a wolf sample, and phylogenetic analysis of the sequence showed that the wolf bocavirus identified formed a cluster distinct from other known canine bocaviruses. To expand our knowledge on this undescribed virus epidemiology, we screened wolf fecal samples using specific PCR primers for this undescribed bocavirus strain. We observed that in one of the wolves, the bocavirus could be detected every year from 2009 to 2012. Also of interest, several wolves belonging to the same pack were positive for the virus at the same time (Table 2 and S1). In dogs, the pathogenicity of bocaviruses is better studied for canine minute virus (CnMV), but less elucidated for the more recently identified bocaviruses. However, a recent fatal outbreak of enteritis in dogs was attributed to a canine bocavirus (Bodewes et al., 2014). Overall, this suggests that bocaviruses have the potential to cause disease, but we cannot exclude that under certain circumstances, this virus could be just an asymptomatic transient passenger, or even derived from an infected prey. These data provide novel insights on this agent, which is not standardly screened for. These results underline that this undescribed virus might be circulating among populations, and it would be a candidate to be added to the panel of screening agents for diarrhea.

Canine minute virus (CnMV) has been identified in healthy dogs but also has been associated with mortality of puppies and elderly dogs (Decaro et al., 2012; Jarplid, Johansson, & Carmichael, 1996; Ohshima, Kawakami, Abe, & Mochizuki, 2010). Serological studies indicate that CnMV is rather widespread in domestic dogs (Jang et al., 2003; Mochizuki et al., 2002). We identified a complete genome of a CnMV in a pet dog diarrheic fecal sample. Due to the high percentage of CnMV reads and the absence of other eukaryotic viral reads in the sample, we can speculate that the virus could have played a role in the pet dog gastrointestinal disease. However, this remains to be further elucidated and it highlights the current challenge to associate viruses with disease.

Moreover, we identified viruses that are likely associated with diet, which is the case of the caninovirus, gemycircularviruses, and densoviruses. In fact, even though the caninovirus was isolated from a feral dog, it is likely of insect origin (as the remaining members of the *Alphanodavirus* genus, family *Nodaviridae*). This is plausible, as canids may prey on insects and feed on carcasses, which are often infested with a wide range of insects. We also cannot exclude the fact that fecal samples could have been colonized by insects after deposition. However, due to the lack of complete genomes and large diversity of alphanodaviruses, it still remains a challenge to identify the true host species of these viruses. Similarly, the identified gemycircularviruses and densoviruses in wolves are also likely of dietary origin. Further studies are needed to elucidate their putative origin and host species.

This study provides a better understanding on the viral populations in the gut of canids, as well as the cocirculation of various known pathogens in both wolves and dogs. While the genetic similarity between domestic and wild canid hosts may play a role in viral host switching, the intensity and rate of contact are critical in this process (Parrish et al., 2008). Hence, our findings raise awareness for the need for a thorough viral screening among wild canid populations for conservation purposes, particularly the ones occurring in anthropogenically modified environments in close contact with domestic dogs. We detected CDV, a well‐known canid pathogen which has been proven to cause mortality and seriously compromise conservation efforts (Di Sabatino et al., 2014; Mech et al., 2008; Müller et al., 2011; Wilmers et al., 2006). Interestingly, we also identified a novel bocavirus complete genome in wolf feces, with unknown pathogenic implications.

In this study, we aimed to study viruses using an established technique (Conceição‐Neto, Zeller, Heylen, et al., 2015; Conceição‐Neto, Zeller, et al. 2016), which has been assessed for its reproducibility and performance. Using this approach, we have focused on viral populations in the gut of canids, not taking into account bacteria or other members of the gut microbiota. Efficient viral enrichment techniques combined with next‐generation sequencing allow researchers to investigate the existence of intestinal viral disease complexes, by not only focusing on the classical viral pathogens, but rather targeting unknown or putative emerging viruses. Moreover, sampling in this study relied on scat surveys genetically validated which are the most commonly used method to monitor elusive carnivores at low densities and thus prone to provide a reasonable and reliable source of noninvasive samples for disease surveillance. These new approaches will change the way pathogens will be detected in the future and might contribute to increasing the panel of virus subjects to be standard screening on studies focusing on wildlife diseases.

## Conflict of Interest

None declared.

## Authors' Contributions

NCN, RG, FA, JRM, MVR, and JM conceived the ideas and designed methodology. SR and FPF collected the samples and field data. NCN, CKY, MZ, and EH performed the NGS sequencing experiments. NCN, CKY, MZ, and JM analyzed the NGS data. NCN, RG, and LL performed the screening and sanger sequencing. NCN, RG, JRM, and JM analyzed the screening data. NCN, WD, and NS prepared the figures. RG, FA, NS, and JRM critically interpreted the viral data in an ecology setting. NCN and JM led the writing of the manuscript. All authors contributed critically to the drafts and gave final approval for publication.
