# Supplementary material for: Viral gut metagenomics of sympatric wild and domestic canids, and monitoring of viruses: Insights from an endangered wolf population
Source: Ecol Evol. 2017 Apr 27;7(12):4135–46. doi: 10.1002/ece3.2991 (PMC5478050; doi:10.1002/ece3.2991)
Supplement: Supplementary file 1 [file ECE3-7-4135-s001.docx]

**Online supporting information**

**
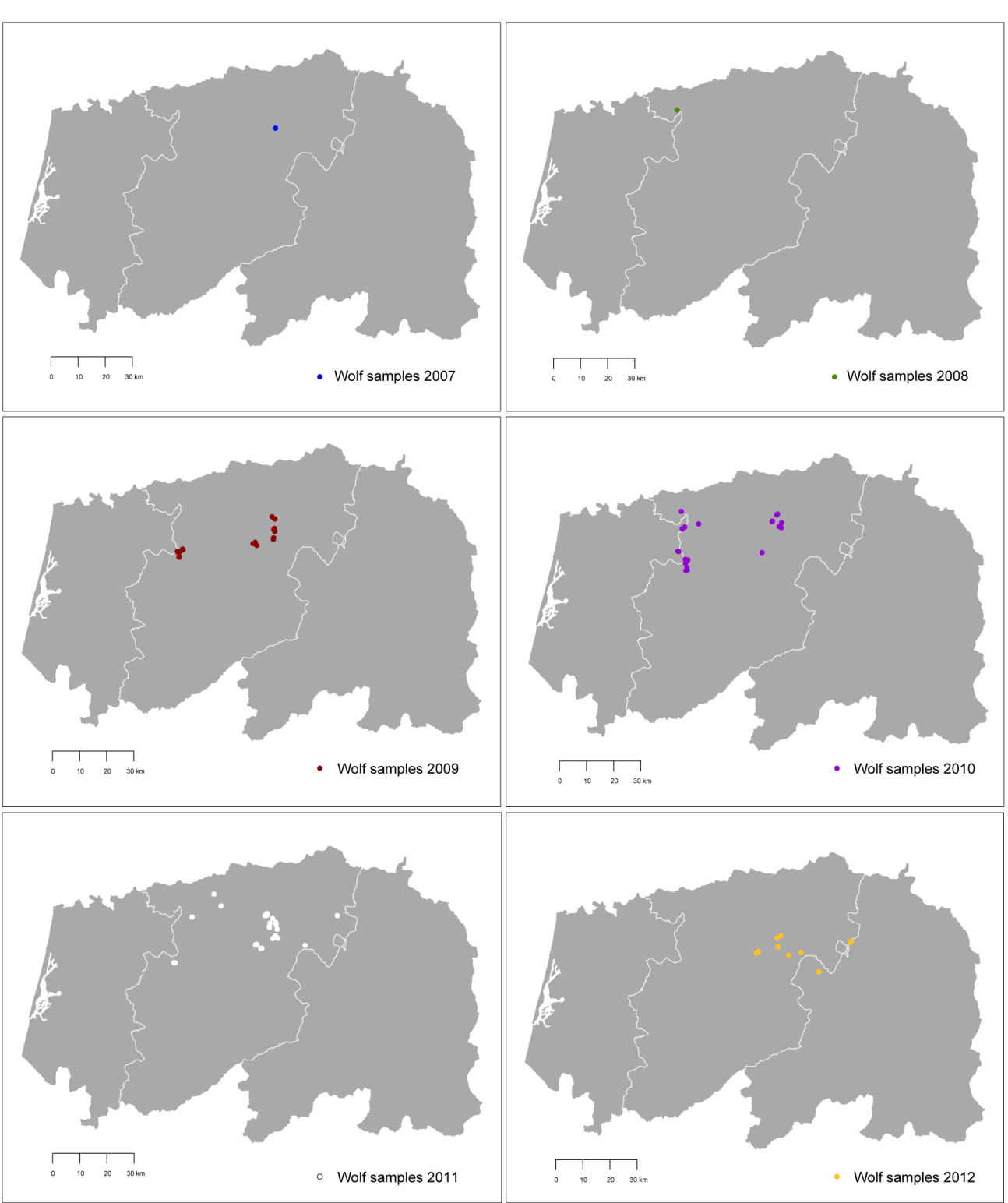
Supplementary figure -** Samples from the PCR screening of endangered wolf pack from the South of Douro region per year (2009-2012).

**Supplementary table –** Information details (per pack) of wolf faecal samples analysed by PCR screening collected in six packs comprising the endangered wolf population at South of Douro river.

| **Pack** | **Sample collection** | **Gauss X** | **Gauss Y** | **Wolf ID** | **Bocavirus** | **CDV** |
| --- | --- | --- | --- | --- | --- | --- |
| Arada | 01/03/09 | 204290 | 432977 | LSD03 | Y | N |
| Arada | 06/07/09 | 202874 | 430300 | LSD03 | N | N |
| Arada | 06/07/09 | 202869 | 430534 | LSD03 | N | N |
| Arada | 08/07/09 | 202307 | 432242 | LSD03 | N | N |
| Arada | 03/12/09 | 203144 | 432353 | LSD03 | Y | N |
| Arada | 03/12/09 | 202302 | 432250 | LSD03 | Y | N |
| Arada | 03/12/09 | 204155 | 433254 | LSD03 | N | N |
| Arada | 04/03/10 | 202670 | 431652 | LSD03 | Y | N |
| Arada | 04/03/10 | 200167 | 435931 | LSD03 | Y | N |
| Arada | 18/04/10 | 203512 | 432864 | LSD10 | N | N |
| Arada | 07/07/09 | 202841 | 431947 | LSD14 | Y | N |
| Arada | 03/12/09 | 202850 | 432005 | LSD14 | N | N |
| Arada | 03/12/09 | 204137 | 433272 | LSD14 | N | N |
| Arada | 26/01/11 | 200773 | 429031 | LSD14 | N | N |
| Arada | 02/06/09 | 202261 | 432447 | LSD24 | N | N |
| Arada | 11/02/10 | 202570 | 433102 | LSD35 | Y | N |
| Arada | 04/03/10 | 203292 | 429176 | LSD35 | N | N |
| Arada | 07/04/10 | 203168 | 430023 | LSD35 | Y | N |
| Arada | 07/04/10 | 202835 | 428858 | LSD35 | Y | N |
| Arada | 21/05/10 | 199854 | 436127 | LSD35 | Y | N |
| Arada | 20/02/11 | 201331 | 429038 | LSD42 | N | N |
| Cinfães | 20/03/11 | 207483 | 445786 | LSD25 | N | Y |
| Cinfães | 16/09/10 | 207448 | 446038 | LSD36 | N | N |
| Cinfães | 15/10/10 | 201469 | 444280 | LSD36 | N | N |
| Cinfães | 18/12/10 | 201128 | 450647 | LSD36 | N | N |
| Cinfães | 12/12/10 | 202344 | 444912 | LSD37 | Y | N |
| Lapa | 17/07/12 | 252950 | 428297 | LSD33 | Y | N |
| Lapa | 29/05/11 | 248807 | 435577 | LSD41 | N | N |
| Lapa | 16/06/12 | 246375 | 435292 | LSD41 | Y | N |
| Lapa | 17/07/12 | 252965 | 428235 | LSD41 | Y | N |
| Lapa | 17/04/12 | 241836 | 434235 | LSD49 | N | N |
| Leomil | 20/06/11 | 235632 | 442388 | Demo | N | N |
| Leomil | 29/10/11 | 232809 | 434371 | Demo | N | N |
| Leomil | 07/07/11 | 230698 | 435568 | LSD07 | Y | N |
| Leomil | 30/10/11 | 234506 | 446879 | LSD07 | N | Y |
| Leomil | 10/12/11 | 232547 | 434453 | LSD07 | N | N |
| Leomil | 12/12/11 | 231139 | 435889 | LSD07 | Y | Y |
| Leomil | 25/11/09 | 230724 | 435801 | LSD13 | N | N |
| Leomil | 14/12/09 | 229959 | 435347 | LSD13 | N | N |
| Leomil | 14/01/10 | 230688 | 435628 | LSD13 | N | N |
| Leomil | 19/09/09 | 237516 | 440519 | LSD16 | N | N |
| Leomil | 23/12/09 | 237890 | 439849 | LSD16 | N | N |
| Leomil | 02/10/09 | 237494 | 437469 | LSD17 | N | N |
| Leomil | 09/10/09 | 236903 | 445175 | LSD17 | N | N |
| Leomil | 05/02/09 | 231332 | 434692 | LSD19 | N | N |
| Leomil | 30/08/09 | 237744 | 440897 | LSD21 | Y | N |
| Leomil | 03/03/10 | 236071 | 449421 | LSD21 | Y | N |
| Leomil | 22/03/11 | 230733 | 435812 | LSD21 | N | N |
| Leomil | 27/10/11 | 237019 | 438061 | LSD21 | Y | N |
| Leomil | 23/10/09 | 237406 | 436930 | LSD22 | Y | N |
| Leomil | 25/02/10 | 237702 | 444759 | LSD22 | Y | Y |
| Leomil | 20/07/10 | 237836 | 446598 | LSD22 | N | N |
| Leomil | 14/01/11 | 238138 | 443062 | LSD22 | Y | N |
| Leomil | 11/08/11 | 238164 | 443156 | LSD22 | N | N |
| Leomil | 10/12/11 | 238873 | 438144 | LSD22 | Y | N |
| Leomil | 13/12/11 | 235943 | 443688 | LSD22 | Y | N |
| Leomil | 28/01/12 | 237499 | 440515 | LSD22 | Y | N |
| Leomil | 21/04/12 | 238802 | 441476 | LSD22 | Y | N |
| Leomil | 18/12/09 | 237900 | 444375 | LSD23 | N | N |
| Leomil | 03/03/10 | 236049 | 449437 | LSD23 |  | N |
| Leomil | 03/03/10 | 236285 | 449734 | LSD23 | N | N |
| Leomil | 03/03/10 | 234447 | 447170 | LSD23 | N | Y |
| Leomil | 03/03/10 | 236072 | 449428 | LSD23 | N | N |
| Leomil | 14/12/10 | 236911 | 445178 | LSD23 | N | N |
| Leomil | 17/01/11 | 238540 | 441458 | LSD38 | Y | N |
| Leomil | 23/04/11 | 234100 | 446387 | LSD38 | N | N |
| Leomil | 03/03/10 | 234356 | 446997 | LSD39 | N | N |
| Leomil | 19/01/11 | 238029 | 443961 | LSD40 | Y | N |
| Leomil | 23/05/11 | 234895 | 447164 | LSD40 | N | Y |
| Leomil | 15/06/11 | 232698 | 434402 | LSD40 | Y | N |
| Leomil | 20/06/11 | 238544 | 441459 | LSD40 | Y | N |
| Leomil | 20/01/11 | 236901 | 445356 | LSD41 | N | N |
| Leomil | 21/04/12 | 238948 | 441459 | LSD43 | N | N |
| Leomil | 23/04/11 | 234579 | 446755 | LSD44 | Y | Y |
| Leomil | 20/06/11 | 235734 | 442082 | LSD44 | N | N |
| Leomil | 07/07/11 | 233031 | 434426 | LSD44 | Y | N |
| Leomil | 24/04/11 | 230746 | 435816 | LSD45 | N | Y |
| Leomil | 11/08/11 | 238107 | 443152 | LSD46 | N | N |
| Leomil | 20/06/11 | 238545 | 441465 | LSD47 | N | N |
| Leomil | 15/06/11 | 230967 | 435887 | LSD49 | N | N |
| Leomil | 23/04/11 | 234906 | 446563 | LSD50 | N | N |
| Leomil | 23/04/12 | 230696 | 435566 | LSD52 | N | N |
| Leomil | 12/12/11 | 237982 | 439057 | LSD53 | Y | N |
| Leomil | 22/05/12 | 237886 | 437357 | LSD53 | N | N |
| Leomil | 09/07/12 | 229941 | 435009 | LSD53 | N | N |
| Leomil | 09/07/12 | 230039 | 435082 | LSD53 | Y | N |
| Leomil | 09/07/12 | 230704 | 435436 | LSD53 | N | N |
| Montemuro | 11/01/11 | 218030 | 449869 | LSD12 | N | N |
| Montemuro | 23/02/11 | 215459 | 454206 | LSD43 | N | N |
| Trancoso | 01/05/11 | 260502 | 446535 | LSD48 | N | N |
| Trancoso | 13/01/12 | 264357 | 439117 | LSD51 | N | N |
| Trancoso | 19/07/12 | 264388 | 439164 | LSD54 | N | Y |
| Trancoso | 19/07/12 | 264693 | 439617 | LSD54 | N | N |
